# Raman spectroscopic detection of high-grade cervical cytology: Using morphologically normal appearing cells

**DOI:** 10.1038/s41598-018-33417-8

**Published:** 2018-10-09

**Authors:** Shiyamala Duraipandian, Damien Traynor, Padraig Kearney, Cara Martin, John J. O’Leary, Fiona M. Lyng

**Affiliations:** 1 DIT Centre for Radiation and Environmental Science, Focas Research Institute, Dublin Institute of Technology (DIT), Camden Row, Dublin, 8 Ireland; 2 School of Physics, Dublin Institute of Technology (DIT), Camden Row, Dublin, 8 Ireland; 3 Department of Pathology, Coombe Women & Infants University Hospital, Dublin, Ireland; 4 Discipline of Histopathology, Trinity College, Dublin, Ireland; 5 Danish Fundamental Metrology, Kogle Alle 5, 2970 Hørsholm, Denmark

## Abstract

This study aims to detect high grade squamous intraepithelial cells (HSIL) by investigating HSIL associated biochemical changes in morphologically normal appearing intermediate and superficial cells using Raman spectroscopy. Raman spectra (n = 755) were measured from intermediate and superficial cells from negative cytology ThinPrep specimens (n = 18) and from morphologically normal appearing intermediate and superficial cells from HSIL cytology ThinPrep specimens (n = 17). The Raman data was subjected to multivariate algorithms including the standard principal component analysis (PCA)-linear discriminant analysis (LDA) and partial least squares discriminant analysis (PLS-DA) together with random subsets cross-validation for discriminating negative cytology from HSIL. The PCA-LDA method yielded sensitivities of 74.9%, 72.8%, and 75.6% and specificities of 89.9%, 81.9%, and 84.5%, for HSIL diagnosis based on the dataset obtained from intermediate, superficial and mixed intermediate/superficial cells, respectively. The PLS-DA method provided improved sensitivities of 95.5%, 95.2% and 96.1% and specificities of 92.7%, 94.7% and 93.5% compared to the PCA-LDA method. The results demonstrate that the biochemical signatures of morphologically normal appearing cells can be used to discriminate between negative and HSIL cytology. In addition, it was found that mixed intermediate and superficial cells could be used for HSIL diagnosis as the biochemical differences between negative and HSIL cytology were greater than the biochemical differences between intermediate and superficial cell types.

## Introduction

Cervical cancer is the 4^th^ most common cause of cancer death in women worldwide and the 7^th^ most common cause of cancer death in females in Europe^1^. In 2012, more than 265,000 deaths were estimated from cervical cancer worldwide^1^. Persistent infection with human papilloma virus (HPV) causes almost all cervical cancer and precancer. The Papanicolaou (Pap) test is the routine test for identifying any abnormality in the cervix. In the Pap test, the cells are scraped from the cervix and examined for the presence of precancerous and cancerous changes. The Pap test results have the following categories: negative for neoplastic cellular changes, atypical squamous cells of undetermined significance, low-grade squamous intraepithelial lesions (LSIL) and high-grade squamous intraepithelial lesion (HSIL)^2^. The identification of cancer and precancer using this standard cytology method depends on the visual assessment of characteristics of individual cells^3^. This can cause intra- and inter-observer disagreements and makes this standard cytology procedure highly subjective with very low sensitivity (68.5%)^4^. Hence, there is a high possibility for missing the premalignant lesions of the cervix. Currently, there is an unmet clinical need to develop new methods to objectively identify patients who are at high risk of developing cervical cancer.

Recently, Raman spectroscopy has received huge interest due to its capability to probe the fingerprint of biomolecules and their changes associated with cancer and its precursors^3,5–9^. Numerous *ex vivo* and *in vivo* studies have explored the potential of Raman spectroscopy for cervical cancer and precancer detection in tissues and cell lines^6,7,9–13^. Relatively few studies have focused on cervical cytology^9,13–16^. Using cell pellets rather than single cells, Vargis *et al*.^15^ showed that HPV-positive and -negative cytology samples could be classified with an accuracy of 98.5% and Rubina *et al*.^14^ showed that normal and cervical cancer cytology samples could be classified with an accuracy of ~80%. Previous work from our group has developed a protocol for processing liquid-based cytology (ThinPrep) cervical specimens for Raman spectroscopic analysis^16^. High quality Raman spectra were successfully recorded from single exfoliated cells and >90% sensitivity and specificity was achieved to discriminate the cells with negative cytology from the cells with HSIL cytology^13,17^. In these cervical cytology samples, the majority of the cells are from uppermost layers of cervical epithelium (i.e., intermediate and superficial layers), while parabasal cells (from the basal layer) are more abundant in atrophic smears from menopausal women. A recent study from our group has shown that each cell type (superficial, intermediate, parabasal) has its own unique Raman signatures which can be discriminated from the Raman signatures of HSIL cells^13^. As cervical precancer progresses from basal to superficial layer, the morphological features/changes associated with HSIL may not be apparent in the uppermost intermediate and superficial layers. Furthermore, Raman spectroscopy requires unstained slides for investigation of cells and tissues. Consequently, it can be extremely difficult to find the rare HSIL cells (morphologically abnormal cells) in these unstained ThinPrep slides. In an early Fourier-transform infrared (FTIR) spectroscopy study, Cohenford and Rigas showed that the spectra of morphologically normal cells from women with dysplasia or cancer exhibit extensive IR spectroscopic changes^18^. This finding was later confirmed by Schubert *et al*. who showed that FTIR spectral changes in cytologically normal cells are most likely due to HPV infection^19^. In the present study, the initial aim was to determine if it was possible to detect HSIL related changes in the cervix by analysing the morphologically normal appearing superficial and intermediate epithelial cells in HSIL cytology samples using Raman spectroscopy. To our knowledge, this has not been shown previously. A further aim was to determine the ability to discriminate between the negative and HSIL cytology cases using intermediate cells, superficial cells and a mixed population of intermediate and superficial cells.

## Materials and Methods

### Sample collection and processing

Negative and HSIL ThinPrep cytology specimens were collected from the colposcopy clinic, Coombe Women and Infants University Hospital (CWIUH), Dublin, Ireland. This study was approved by and carried out in accordance with the Research Ethics Committee at CWIUH. After obtaining informed consent, the specimens were collected according to cytology standard operating procedures (SOP) and then processed via ThinPrep^TM^ method^16^. The cells from the cervix were scraped using a cytobrush and then rinsed in the specimen vial containing a methanol based fixative i.e., PreservCyt transport medium (ThinPrep Pap Test, Screenlink, Dublin). Although fixation and other processing steps have been shown to result in biochemical changes in cells and tissues^20^, it was not possible to change this procedure as these were clinical samples collected according to the standard cytology laboratory workflow. The labelled ThinPrep sample vials were then sent to the cytology laboratory equipped with a ThinPrep processor. The ThinPrep processor (Hologic Inc., Marlborough, MA) homogenizes the sample by spinning the filter (T2000), creating shear forces in the fluid that are strong enough to disaggregate randomly joined material, break up blood, mucus and non-diagnostic debris while keeping true cell clusters intact^16^. The cells were then collected onto the membrane of the TransCyt filter and further transferred onto a glass slide to create a monolayer deposit of cells (~20 mm in diameter). The slide was then transferred into a fixative bath of 95% ethanol automatically. In total, 35 unstained cytology samples on ThinPrep^TM^ slides (18 negative and 17 HSIL) were obtained and subjected to Raman spectroscopic analysis. Before Raman measurement, each slide was pre-treated with hydrogen peroxide (H_2_O_2_) to remove any contaminating blood and debris^16^. Briefly, the slides were treated with a 30% solution of H_2_O_2_ at room temperature for 3 minutes. The slides were then placed into a 70% solution of industrial methylated spirits (IMS) for 3 minutes followed by multiple dips into 100% IMS to remove any remaining cellular debris and H_2_O_2_. The slide was then air dried.

### Raman instrumentation

Raman spectra were recorded using a HORIBA Jobin Yvon XploRA^TM^ system (Villeneuve d’Ascq, France), incorporating an Olympus microscope BX41 equipped with a ×100 objective (NA = 0.9). The system consists of a 532 nm diode laser, 1200 lines/mm grating and an air-cooled CCD detector (024 × 256 pixels). The system was wavelength calibrated to the 520.7 cm^−1^ spectral line of silicon and also intensity-calibrated using a relative intensity correction standard (NIST 2242). In this study, a total of ~755 Raman signals were measured from the ThinPrep specimens of 35 patients (18 negative and 17 HSIL). From each slide, 10 to 15 intermediate and superficial epithelial cells were randomly selected and good quality Raman spectra were obtained with an integration time of 30 sec and 2 accumulations to improve the signal to noise ratio. The laser power on the sample was ~1 mW. The images of the Raman measured cells were recorded together with x- and y- coordinates. After the Raman spectral acquisition, the samples were Pap stained and each recorded cell was re-visited using the stored x- and y- co-ordinates to verify whether the cells were from the intermediate or superficial layer.

### Data analysis

All the recorded Raman spectra were corrected for the glass background using a linear least-squares method with non-negative constraints. The least-squares model was developed using the basis spectra obtained from pure glass slides and selected pure biochemicals (e.g., actin, collagen, RNA, DNA, etc.) that approximate the biochemical composition of cervical cells. The Raman dataset has also been corrected for the baseline and then vector normalized. The Raman data was mean-centered and then subjected to multivariate algorithms including the standard principal component analysis (PCA)-linear discriminant analysis (LDA)^21^ and partial least squares discriminant analysis (PLS-DA)^22,23^ together with random subsets cross-validation for discriminating negative cytology from HSIL. In this cross validation, the data are randomly split into many subsets. Different test sets are selected through random selection of samples (total number of samples/number of split (n = 20)) in the dataset in such a way that no single sample is in more than one test set and this procedure is iterated multiple times. PCA projects the data in the direction of maximum variability and retains most of the information in a smaller number of these projected variables. LDA further maximizes separation between the classes in these PCA-projected variables and minimizes the separation within a class^21^. Following PCA-LDA, PLS-DA was applied on the same dataset because PCA only involves one set of data, but PLS reduces the data by explaining as much variance as possible by considering the correlated relationships between the Raman spectral dataset and the class membership (i.e., 0 s and 1 s to represent each observation)^24,25^. Hence, PLS-DA considers diagnostically relevant variations and obtains maximum group separation by rotating the latent variables (LVs). The PCA-LDA was carried out using custom scripts written in MATLAB (Mathworks, Inc. Natick, MA). The PLS-DA analysis was performed using the PLS toolbox (Eigenvector Research, Wenatchee, WA) in the Matlab scripting environment.

## Results and Discussion

**Figure 1:**
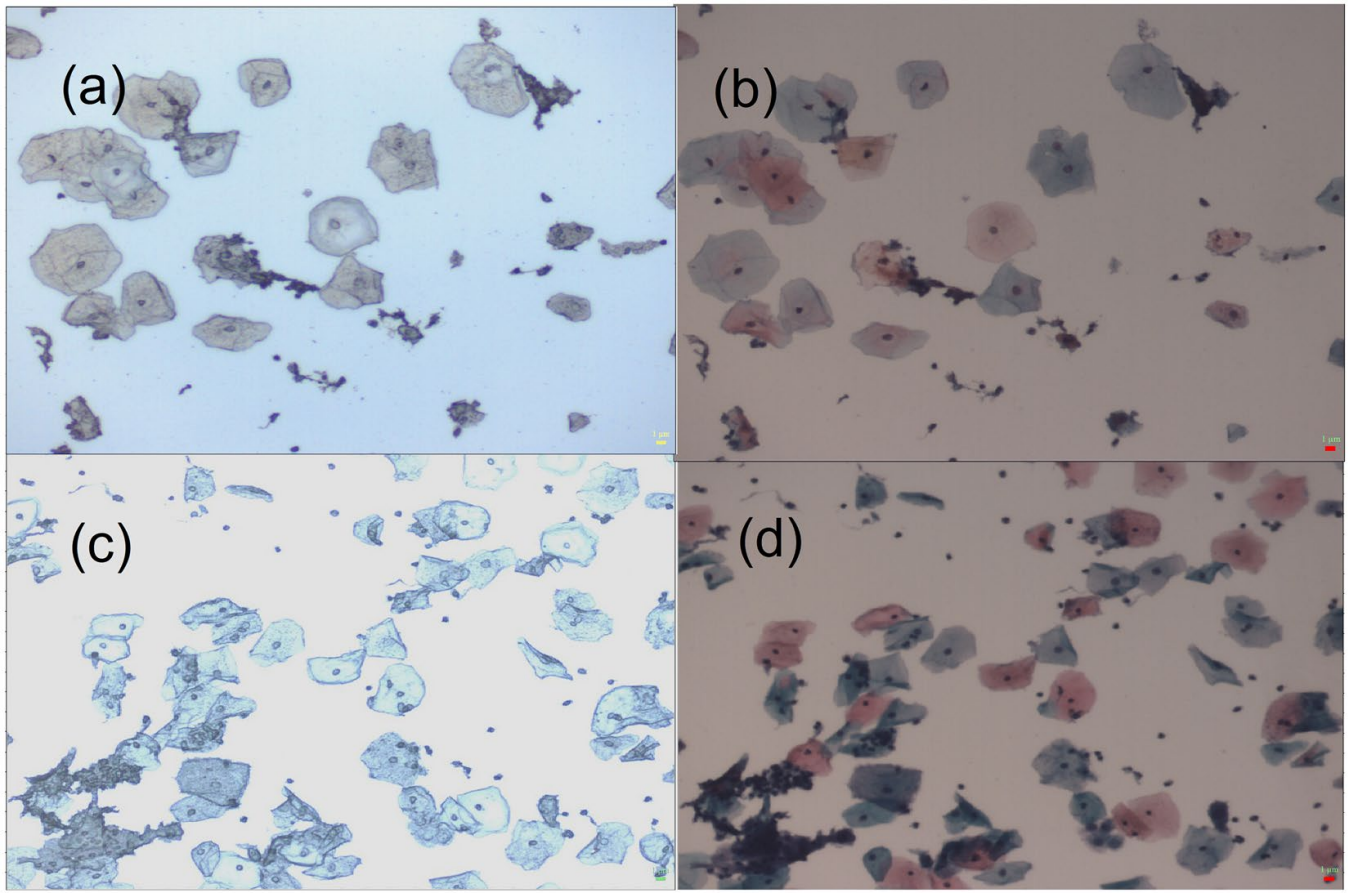
(**a**) Unstained and (**b**) Pap stained image of negative cytology specimen, X10. (**c**) Unstained and (**d**) Pap stained image of high-grade squamous intraepithelial lesion (HSIL) cytology specimen, X10. In (**b**,**d**), superficial cells are orange to pink in colour and intermediate cells are turquoise green to blue in colour.

Figure 1 shows the intermediate and the superficial cells collected from the negative and HSIL cytology specimens. The intermediate and the superficial cells are indistinguishable in unstained slides (Fig. 1(a,c)). Raman spectra were randomly recorded from the morphologically normal looking cells from unstained negative and HSIL cytology slides as the Pap stain components have their own unique Raman signatures which interfere with the cellular Raman spectrum. Following the Raman measurements, the x, y coordinates for each cell were recorded. After completing the Raman measurements, the slide was stained using the Pap stain. Using the recorded x, y coordinates, each cell was reviewed and assigned as intermediate or superficial based on the staining pattern i.e., the intermediate cells are turquoise green to blue in colour and the superficial cells are orange to pink in colour (Fig. 1(b,d)).

**Figure 2:**
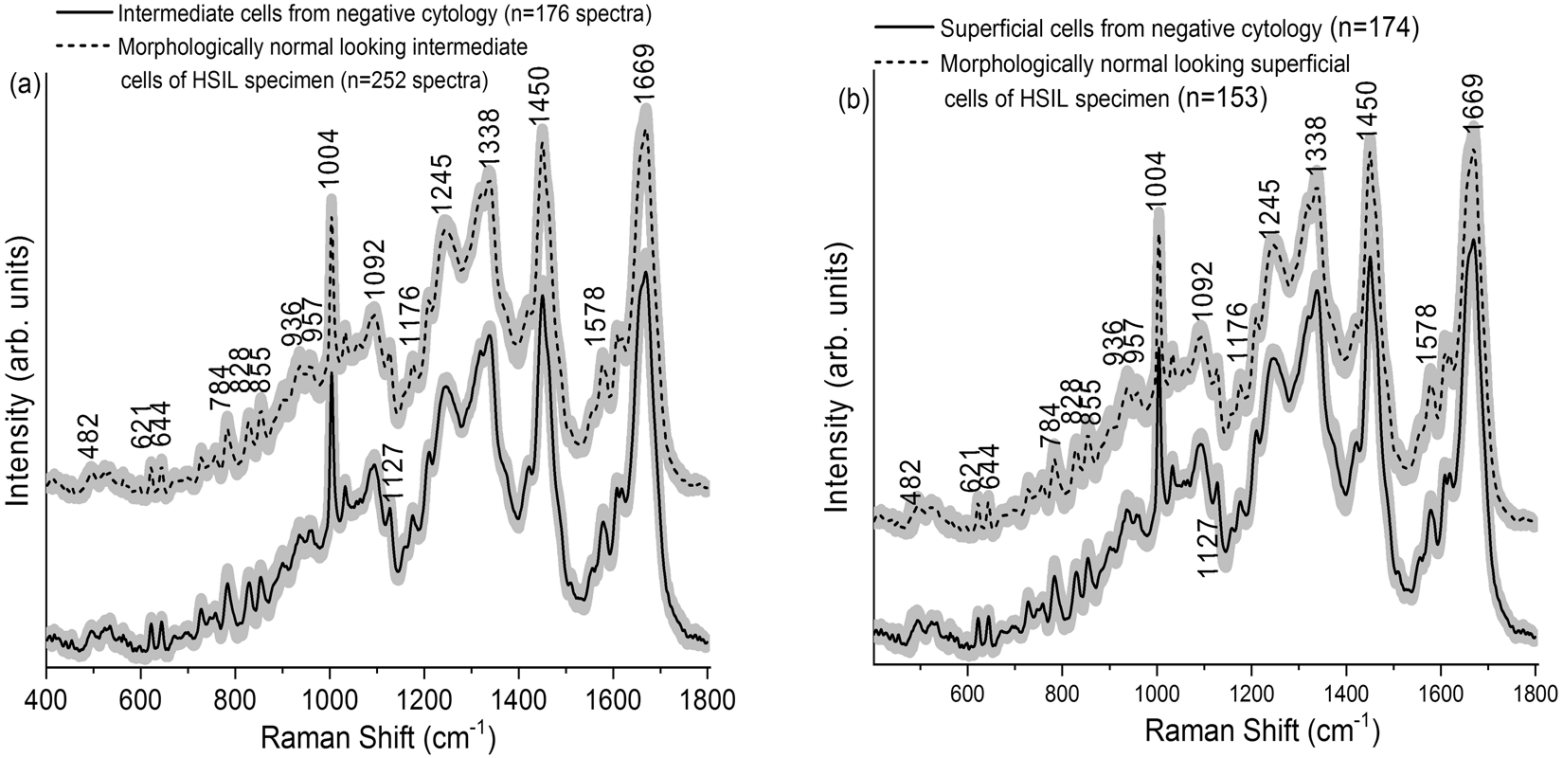
(**a**) Mean Raman spectra ±1 standard deviation (SD) acquired from the intermediate cells of negative cytology ThinPrep specimens (n = 18) and morphologically normal appearing intermediate cells of high-grade squamous intraepithelial lesion (HSIL) ThinPrep specimens (n = 17). (**b**) Mean Raman spectra ±1 SD acquired from the superficial cells of negative cytology specimens (n = 18) and morphologically normal appearing superficial cells of HSIL cytology ThinPrep specimens (n = 17).

**Figure 3:**
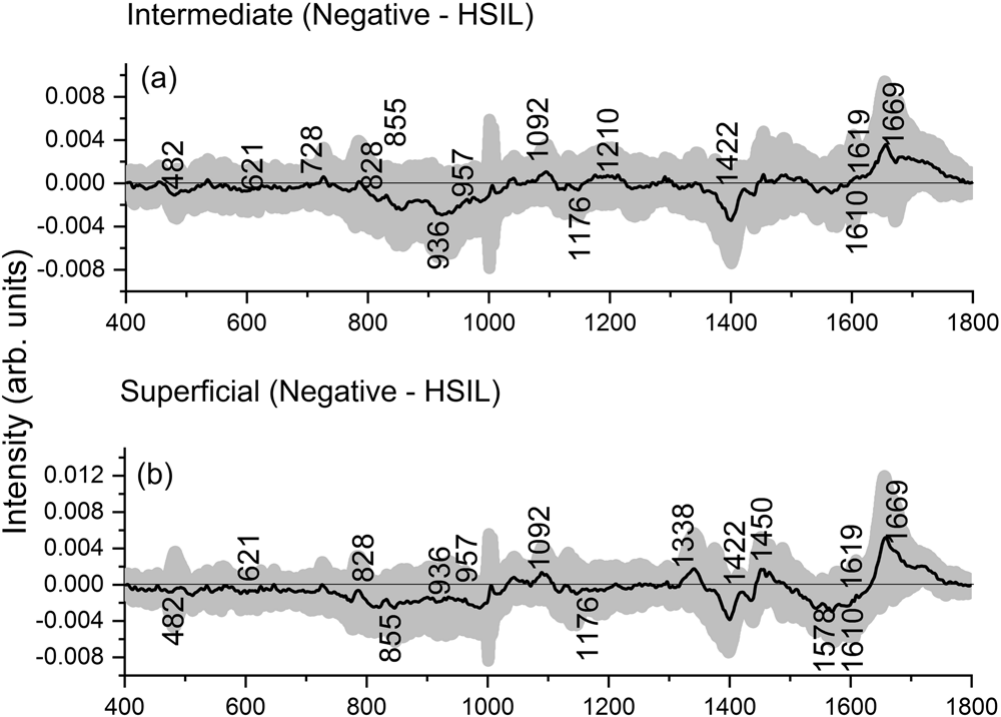
Difference Raman spectra ± 1 standard deviation (SD) obtained from (**a**) intermediate cells of negative cytology and high-grade squamous intraepithelial lesion (HSIL) cytology specimens and (**b**) superficial cells of negative cytology and HSIL cytology specimens. Raman spectra were acquired from morphologically normal appearing intermediate and superficial cells in the HSIL cytology specimens.

The mean normalized Raman spectra obtained from the intermediate cells (negative (n = 176), HSIL (n = 252), Fig. 2a) and the superficial cells (negative (n = 174), HSIL (n = 153), Fig. 2b) are shown in Fig. 2. The spectra were recorded in the fingerprint region (400 to 1800 cm^−1^) due to the presence of abundant Raman bands that are unique for different biomolecules that comprise cervical cells and tissue, such as proteins, glycogen, lipids, DNA, etc. The Raman spectra obtained from the intermediate cells (maximum standard deviation (SD) = ±0.0049) and superficial cells (maximum SD = ±0.0056) are quite consistent. A larger spectral variability was reported previously for spectra recorded from the superficial layer in cervical tissue sections due to the variation in the glycogen content associated with women’s age, hormonal levels and menstrual cycle^26^. Our previous study on cervical exfoliated cells also showed a higher spectral variability in the spectra recorded from the cytoplasm due to glycogen content^13^. In the present study, the consistency observed among the Raman spectra of superficial cells is probably due to the reason that the Raman signals were measured from the cell nucleus. The measured Raman spectra from intermediate and superficial cell types contain complex, overlapped spectral signatures in relation to the tissue biochemistry. Raman peaks can be observed in the vicinity of 482 cm^−1^ (glycogen), 621 and 644 cm^−1^ (proteins), 784 cm^−1^ (DNA), 828 cm^−1^ (DNA/RNA), 855 and 936 cm^−1^ (glycogen and proteins), 957 cm^−1^ (DNA), 1004 cm^−1^ (phenylalanine), 1092 cm^−1^ (DNA phosphate backbone), 1127 cm^−1^ (proteins), 1176 cm^−1^ (cytosine/guanine), 1245 cm^−1^ (amide III), 1338 cm^−1^ (proteins and nucleic acids), 1450 cm^−1^ (proteins, lipids), 1578 cm^−1^ (nucleic acids), and 1669 cm^−1^ (amide I)^3,6,16,17,27–29^. The main Raman peaks and their tentative assignments are summarized in Table 1. The main differences between the Raman spectra acquired from the intermediate cells (Fig. 3a) or superficial cells (Fig. 3b) from negative and HSIL cytology specimens were observed at 482, 621, 728, 828, 855, 936, 957, 1092, 1176, 1210, 1422, 1450, 1578, 1610, 1619, and 1669 cm^−1^ (unpaired two-sided Student’s t-test, p < 0.001). These bands are mainly related to DNA, proteins and glycogen. It is very interesting that many of the features are similar for the intermediate and superficial cells. This can be explained from the fact that the intermediate layer is the transitional layer between the immature cells of the basal/parabasal layer and the mature cells of the superficial layer^30^. Hence, the intermediate and superficial layers are expected to have similar molecular composition such as glycogen, DNA, proteins, etc. This is consistent with our previous study which showed clustering of stroma, basal/parabasal, and superficial/intermediate layers characterised by the spectral features of collagen, DNA bases, and glycogen, respectively^31^.

**Table 1:** Tentative peak assignments for cell Raman spectra displayed in Fig. 2.

Wavenumber (cm^−1^)	Raman Peak Assignments
482	Glycogen
621	C-C twisting mode of Phenylalanine (proteins)
644	C-C twisting mode of Tyrosine and Phenylalanine
784	Uracil, Thymine, Cytosine (ring breathing modes in the DNA/RNA)
828	PO_2_ stretching in DNA, Tyrosine
855	Ring breathing in Tyrosine and Proline (proteins)
936	C-C stretching mode of Proline and Valine
957	C-C and C-N stretch PO_3_^2−^ stretch (DNA)
1004	C-C aromatic ring stretching in Phenylalanine
1092	Symmetric PO_2_^−^ stretching vibration of the DNA
1127	C-N stretching in proteins
1176	C-H in plane bending mode of Tryptophan & Phenylalanine; Cytosine, Guanine
1245	Amide III (of collagen)
1338	CH_2_/CH_3_ wagging & twisting mode in collagen, nucleic acid & tryptophan
1450	CH (CH_2_) bending mode in proteins and lipids
1578	Adenine, Guanine (DNA/RNA); C=C bending mode of Phenylalanine
1669	Amide I (C=O stretching, C-N stretching and N-H bending, proteins)

**Figure 4:**
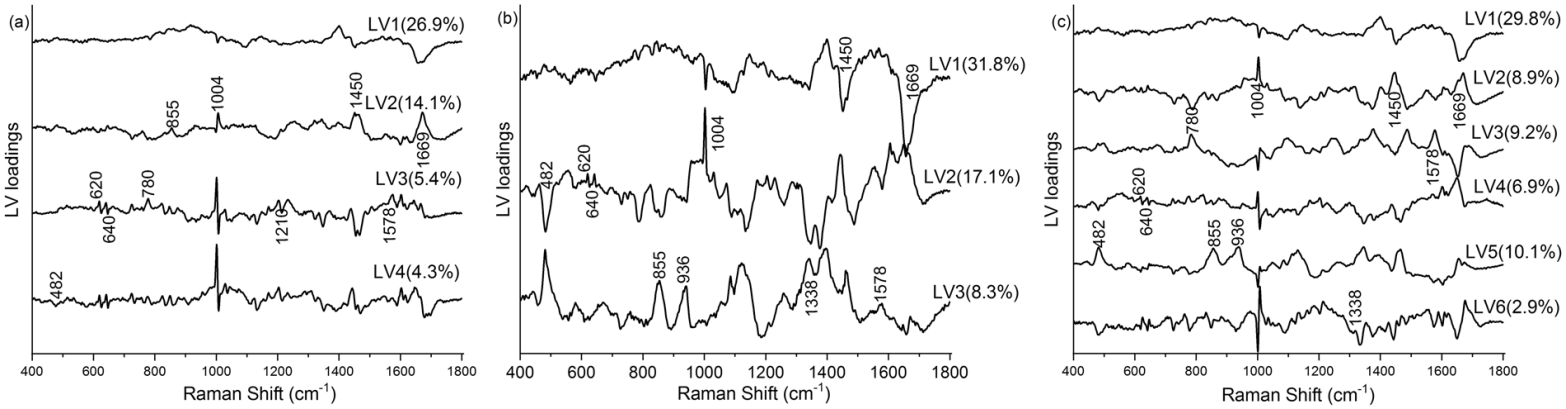
PLS components (LVs) loadings of the developed PLS-DA model for the dataset obtained from (**a**) intermediate cells, (**b**) superficial cells and (**c**) mixed intermediate and superficial cells. (latent variables (LVs), partial least squares discriminant analysis (PLS-DA)).

**Figure 5:**
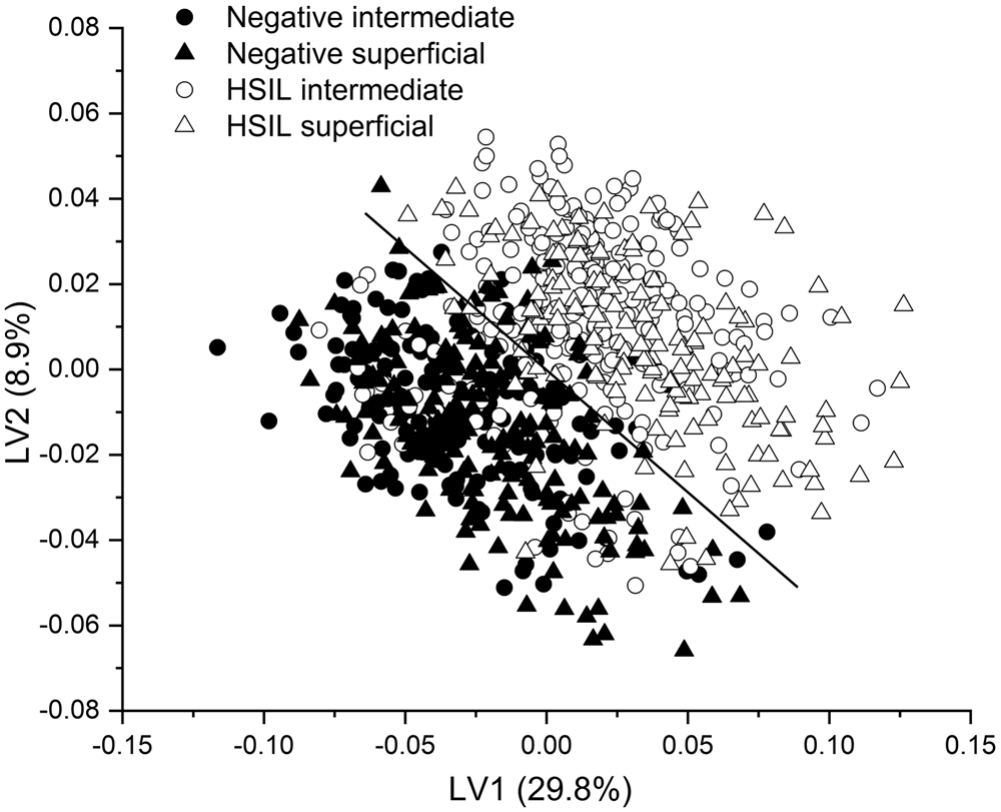
Scatter plot of the significant latent variables (LVs) obtained from the Raman spectral dataset of mixed intermediate and superficial cells of negative cytology and high-grade squamous intraepithelial lesion (HSIL) cytology specimens.

**Figure 6:**
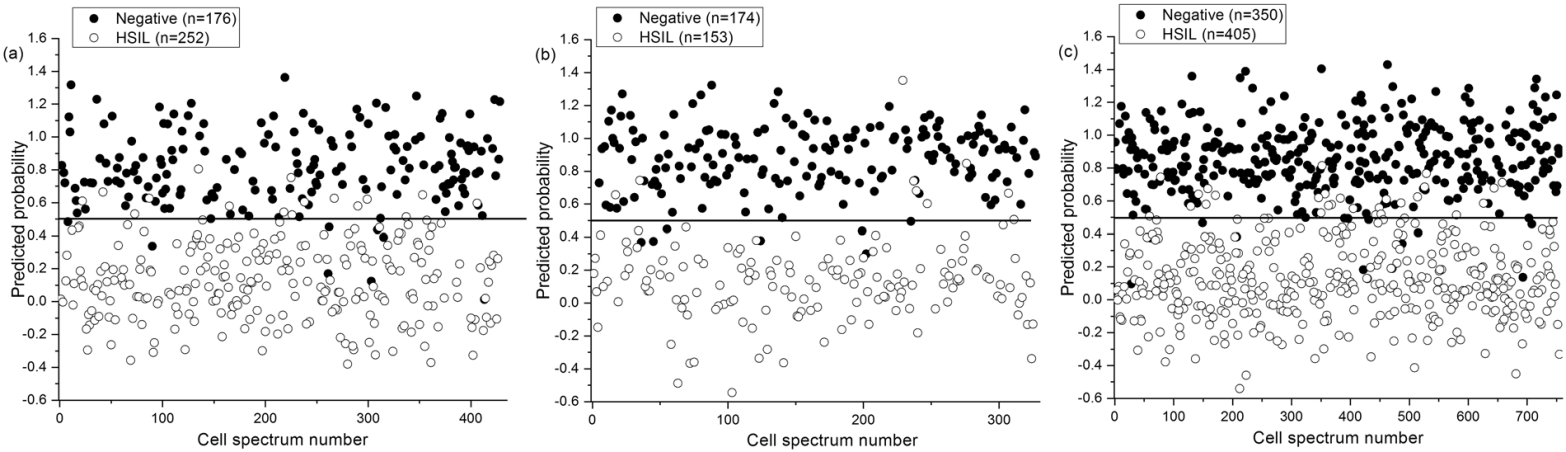
Scatter plot of the predicted probability values calculated from the Raman dataset obtained from (**a**) intermediate cells, (**b**) superficial cells and (**c**) mixed intermediate and superficial cells of negative and HSIL cytology specimens.

As the visual differences between the Raman spectra from negative and HSIL cytology specimens are subtle, multivariate analysis, PLS-DA, was utilized to enhance the spectral differences. The PLS-DA models were developed from the intermediate, superficial, mixed intermediate/superficial dataset. The number of LVs or PLS components (4LVs, 3LVs and 6LVs, Fig. 4) corresponding to minimum cross-validation error were utilized to develop the models for intermediate, superficial and mixed intermediate/superficial datasets, respectively. The LVs (Fig. 4b) for the superficial dataset accounted for 57.2% and 76.5% of the total Raman spectral variations in the X and Y directions and predominantly provided information about DNA and proteins (620, 640, 1004, 1338, 1450, 1578, and 1669 cm^−1^), glycogen and proteins (482, 855, and 936 cm^−1^). Similarly, the LVs (50.7% variation in X direction and 72.3% variation in Y direction, Fig. 4a) corresponding to the intermediate dataset mainly showed changes in glycogen, DNA and protein features (482, 620, 640, 780, 855, 1004, 1210, 1450, 1578, and 1669 cm^−1^). Combining the intermediate and superficial dataset, the LVs (67.8% and 76.0% variations in X and Y direction, Fig. 4c) extracted information around the Raman peaks 780, and 1578 cm^−1^ (DNA), and 482, 620, 640, 855, 936, 1004, 1338, 1450, and 1669 cm^−1^ (glycogen and proteins). From the above results, it is clear that both cell types either utilized separately or in combination provide almost similar diagnostic information associated with HSIL diagnosis. The LV scores scatter plot (Fig. 5) obtained from the mixed intermediate/superficial dataset visually shows that the scores of intermediate and superficial cells from negative cytology specimens are overlapped due to minimal variability between the two cell types. Similarly, the scores of intermediate and superficial cells from HSIL cytology specimens are overlapped. However, the scores of both intermediate and superficial cells showed clear separation between the negative and HSIL cytology specimens. This reinforces the observation that the differences between the negative and HSIL cytology specimens are highly significant compared to the differences between the two cell types. It must be noted that some of the LV scores of the HSIL intermediate dataset are skewed towards the scores of the negative cytology specimens (Fig. 5). This could be attributed to the facts that some HSIL cytology specimens can regress back to normal^32,33^ or some cells in the HSIL specimen can still be normal biochemically as well as morphologically. The predicted probability plots from the developed PLS-DA models provided sensitivities of 95.5%, 95.2% and 96.1% and specificities of 92.7%, 94.7%, and 93.5% (Table 2), respectively, for identifying the HSIL cases based on the spectral dataset obtained from intermediate cells (Fig. 6a), superficial cells (Fig. 6b), and mixed intermediate/superficial cells (Fig. 6c). The predicted probability plot of the mixed intermediate/superficial cells (Fig. 6c) further shows that the diagnostic efficacy of identifying HSIL is not significantly affected by mixing the intermediate and superficial cell types. This result was again consistent with our previous study which showed clustering of stroma, basal/parabasal, and superficial/intermediate layers characterized by the Raman signatures of collagen, DNA bases, and glycogen, respectively^31^. We also compared the diagnostic efficiency of the PLS-DA method with PCA-LDA as this is very frequently used for cancer and precancer diagnosis using Raman spectroscopy. The standard PCA-LDA method provided sensitivities of 74.9%, 72.8%, and 75.6% and specificities of 89.9%, 81.9%, and 84.5%, respectively, for HSIL diagnosis based on the dataset obtained from intermediate, superficial and mixed intermediate/superficial cells. The PLS-DA models were found to perform better than PCA-LDA models because PCA realises the dimensionality reduction by retaining much of the information in the spectral dataset, however, PLS reduces the dimensionality by explaining as much of the variation by considering the correlated relationships between the spectral dataset and the class membership^25^. Since the intermediate and superficial cells can be mixed together without affecting the efficacy of HSIL diagnosis, this tedious and time consuming process of staining and revisiting the Raman measured cells for the identification of cell type can be eliminated in future studies. This study confirms the earlier FTIR spectroscopy studies^18,19^ showing that morphologically normal cells from abnormal samples show distinct biochemical changes and indicate that biochemical changes are pervasive in the epithelium in the presence of a HSIL lesion. These findings support the concept of ‘field change’ in the epithelium at a biochemical/molecular level in cervical precancer. This study has further extended the earlier FTIR spectroscopy studies to demonstrate that HSIL can be detected irrespective of the cell type being measured i.e., intermediate cells, superficial cells or a mixed population of intermediate and superficial cells. Work is ongoing to extend the spectral library of negative and HSIL specimens to improve the PLS-DA model in order to predict unknown specimens in a real clinical setting.

**Table 2:** Calculated sensitivities and specificities for differentiating negative cytology and high-grade squamous intraepithelial lesion (HSIL) cytology using the Raman spectral dataset obtained from (i) intermediate cells, (ii) superficial cells and (iii) mixed intermediate and superficial cells.

Type of cells	PCA-LDA		PLS-DA	
	Sensitivity (%)	Sensitivity (%)	Sensitivity (%)	Specificity (%)
Intermediate	74.9	89.9	95.5	92.7
Superficial	72.8	81.9	95.2	94.7
Intermediate + Superficial	75.6	84.5	96.1	93.5

## Conclusions

Raman spectra were successfully acquired from the intermediate and superficial cells of negative cytology specimens and morphologically normal appearing intermediate and superficial cells of HSIL cytology specimens. The inter-cell type differences in the Raman spectra showed significantly less variability than the differences between the negative and HSIL cytology. This study demonstrates the potential of Raman spectroscopy for identifying HSIL at cytology from the spectra acquired from morphologically normal appearing cells and further shows that a mixed population of superficial and intermediate cell types can be used. It also carries an implication that many cells are potentially committed to a HSIL lineage, although cytologically viewed as normal. Dysplasia therefore can be seen as a cellular ‘bar-coding’ phenomenon and Raman spectroscopy appears to be sensitive enough to detect this. This study brings the translation of Raman spectroscopy to cervical cancer screening closer by eliminating the difficulty of finding the rare morphologically abnormal cells on the unstained slides.

## Data Availability

The datasets generated and analysed during this study are available from the corresponding author on reasonable request.
